# Small firms and the COVID-19 insolvency gap

**DOI:** 10.1007/s11187-021-00514-4

**Published:** 2021-07-06

**Authors:** Julian Oliver Dörr, Georg Licht, Simona Murmann

**Affiliations:** 1 Department of Economics of Innovation and Industrial Dynamics, ZEW Leibniz Centre for European Economic Research, L7 1, 68161 Mannheim, Germany; 2 Department of Econometrics and Statistics, Justus Liebig University Giessen, Licher Straße 64, 35394 Gießen, Germany

**Keywords:** COVID-19, Liquidity support, Insolvency law, Corporate insolvency, Cleansing effect, SMEs, Entrepreneurship, G33, H12, H81, L26

## Abstract

COVID-19 placed a special role on fiscal policy in rescuing companies short of liquidity from insolvency. In the first months of the crisis, SMEs as the backbone of Germany’s economy benefited from large and mainly indiscriminate aid measures. Avoiding business failures in a whatever-it-takes fashion contrasts, however, with the cleansing mechanism of economic crises: a mechanism which forces unviable firms out of the market, thereby reallocating resources efficiently. By focusing on firms’ pre-crisis financial standing, we estimate the extent to which the policy response induced an insolvency gap and analyze whether the gap is characterized by firms which were already struggling before the pandemic. With the policy measures being focused on smaller firms, we also examine whether this insolvency gap differs with respect to firm size. Our results show that the COVID-19 policy response in Germany has triggered a backlog of insolvencies that is particularly pronounced among financially weak, small firms, having potential long-term implications on entrepreneurship and economic recovery.

**Plain English Summary** This study analyzes the extent to which the strong policy support to companies in the early phase of the COVID-19 crisis has prevented a large wave of corporate insolvencies. Using data of about 1.5 million German companies, it is shown that it was mainly smaller firms that experienced strong financial distress and would have gone bankrupt without policy assistance. In times of crises, insolvencies usually allow for a reallocation of employees and capital to more efficient firms. However, the analysis reveals that this ‘cleansing effect’ is hampered in the current crisis as the largely indiscriminate granting of liquidity subsidies and the temporary suspension of the duty to file for insolvency have caused an insolvency gap that is driven by firms which were already in a weak financial position before the crisis. Overall, the insolvency gap is estimated to affect around 25,000 companies, a substantial number compared to the around 16,300 actual insolvencies in 2020. In the ongoing crisis, policy makers should prefer instruments favoring entrepreneurs who respond innovatively to the pandemic instead of prolonging the survival of near-insolvent firms.

## Introduction

COVID-19 and its unprecedented economic impacts have ground economies worldwide to a halt. As a result of the early lockdown measures to contain the spread of the virus, many companies faced reduced business activity and declining sales, which had an immediate impact on their liquidity positions. Indeed, both the negative demand shock paired with a negative supply shock in most industries have put numerous companies under severe pressure to keep their operations afloat. Previous crises have taught that small entrepreneurial firms are particularly prone to considerable liquidity constraints in deep recessions. For example, literature on the financial crisis of 2007–2009 shows that especially small and entrepreneurial enterprises were exposed to a severe liquidity crunch due to the collapse of the interbank market and its negative impact on corporate lending (e.g. Cowling et al., 2012; Iyer et al.^2014^; Lee et al., 2015; McGuinness & Hogan 2016). In the COVID-19 crisis, the early effects of the combined negative supply and demand shock are also characterized by a deep liquidity shock in the real economy. The decline of trading activities and lack of business revenues made many firms dependent on their cash reserves in order to meet their unchanged fixed cost obligations. As smaller and entrepreneurial companies are characterized by strong dependence on internally generated funds to capitalize their business and provide the liquidity needed to finance day-to-day operations, both their cash reserves and collaterals for external financing are generally limited (Cowling et al., 2020). In times of financial distress as in the current COVID-19 crisis, this makes small ventures particularly vulnerable to financial insolvency (Fairlie, 2020; Bartik et al., 2020). Recent research suggests that severely affected small entrepreneurial ventures even seek for alternative financing methods such as bootstrap financing to keep their businesses alive (Block et al., 2021). Trapped in a situation of thin capital reserves and lack of collaterals for drawing new credit lines, small firms face therefore a particularly high risk of business failure without the relief through policy intervention.

Aware of the far-reaching consequences of a wave of corporate insolvencies, governments in almost all countries have initiated a series of emergency measures to strengthen liquidity positions of their national companies, some of which exclusively focusing on easing the burden of Small and Medium-sized Enterprises (SMEs) (OECD, 2020a). In the European Union (EU), for instance, member states’ liquidity support in form of public loan guarantees and tax deferrals for distressed sectors has increased by an estimated 6 percentage points (pp) of EU GDP compared to pre-crisis levels (Council of the European Union, 2020). In most countries, policy measures have gone beyond deferrals and loan guarantees, including instruments such as wage subsidies and adjustments in bankruptcy regimes. While there is no doubt that a strong policy response was necessary to keep the struggling economy afloat, the need to respond quickly and the sheer volume of firms seeking assistance left little time for policymakers to assess the viability of firms that received early government support. Thus, many of the early policy measures were not only unprecedented in scale but also largely granted indiscriminately with the primary focus to avoid corporate bankruptcies.^1^ Even though some programs’ eligibility criteria are formally linked to pandemic-induced financial distress only, information asymmetries make drawing a line often difficult in reality. We argue that these circumstances have favored a substantial backlog of corporate insolvencies as policy measures have also kept otherwise insolvent firms in the market. This phenomenon is referred to as insolvency gap in the remaining of the paper.

The central purpose of this study is to analyze whether the early policy response has indeed induced such an insolvency gap and, if so, by which firms the gap is mainly driven. We do so by incorporating the Schumpeterian cleansing effect usually observed in economic crises into our analysis. In Schumpeterian economics, crises are typically seen as cleansing mechanism forcing unviable firms out of the market thereby efficiently reallocating resources to more productive companies. Our hypothesis is that this cleansing mechanism is strongly compromised by the undifferentiated policy response which favors the survival of otherwise unviable firms. Since in times of crises small firms tend to be particularly prone to liquidity shortages, we believe that the risk of unviable ‘survivors’ is especially high among smaller enterprises. The strong policy focus on SMEs in many countries (OECD, 2020a) reinforces this hypothesis. Finally, it is likely that the prolonged expansion prior to COVID-19 along with the low interest rate environment have already accumulated a substantial number of financially weak companies before the pandemic (Barrero et al., 2020). Normally, the COVID-19 crisis would have been a ‘natural’ mechanism to force such ailing firms out of the market. Given the interplay between prolonged expansion and sudden economic decline paired with a strong policy response, our hypothesis is that the insolvency gap is strongly driven by small firms with weak financial conditions prior to the crisis.

Our contribution to the fast growing literature on the economic effects of the COVID-19 crisis is manifold. First, we examine the heterogeneity with respect to firm size in policy makers’ response to the risk of large-scale business failures. Second, we translate Schumpeter’s theory of the cleansing effect in economic crises into an empirical assessment by estimating the size of a policy-induced insolvency gap using firm-specific credit rating data combined with information on insolvency filings. Controlling for updates in a firm’s credit rating, we estimate the insolvency gap induced by the COVID-19-related policy measures using a potential outcome setting. Based on pre-crisis observations of no policy intervention comparable firms with closely matching changes in their credit rating are used as control group for the estimation of counterfactual insolvency rates. Finally, we discuss the consequences for entrepreneurship if efficient resource reallocation and business liquidation are compromised.

The remainder of the paper proceeds as follows. Section 2 gives an overview of the relevant literature. In Section 3, we discuss the different COVID-19 support instruments for firms in Germany and emphasize their different orientations depending on firm size. Section 4 introduces the data sources and variables used to estimate the insolvency gap. Moreover, the framework for the matching of counterfactual survival states is introduced. Section 5 empirically examines the adverse impacts of the pandemic and its heterogeneity across firms of different size and sector affiliation. Moreover, it presents the empirical results of the insolvency gap estimation. Ultimately, Section 6 discusses the implications of our results and concludes.

## Related literature

The fast growing literature on business failures in response to the adverse economic impacts of COVID-19 stresses that the early assistance packages may bare high economic costs if they keep unviable firms alive (Kalemli-Ozcan et al., 2020; Barrero et al., 2020; Cowling et al., 2020; Juergensen et al., 2020; OECD, 2020b; Didier et al., 2021). Kalemli-Ozcan et al. (2020), for example, find for a number of European countries that without appropriate targeting of policy instruments, the fiscal costs of intervention and the number of ‘ghost’ firms kept alive are substantially higher compared to a scenario in which policies target only ‘viable’ firms. Besides the direct fiscal costs associated with indiscriminate policy interventions, there is yet another source of economic costs associated with keeping unviable firms alive. In Schumpeterian economics, this may also impede the cleansing effect of creative destruction (see, for example, Legrand 2017 and in the COVID-19 context Barrero et al., 2020; Guerini et al., 2020). This effect describes a process in which resources are reallocated from less efficient and less creative firms to more efficient ones enhancing overall economic productivity and innovation (Schumpeter, 1942). Typically, this process of efficient resource reallocation is particularly strong in times of economic crisis, allowing viable and innovative firms to gain market share as unprofitable firms exit the market (Caballero and Hammour, 1994; Archibugi et al., 2013; Carreira & Teixeira, 2016). As such, without the intervention of fiscal policy, business failures of unviable firms are expected to be substantial in economic recessions and, given the strong vulnerability of small and entrepreneurial firms, the effect is expected to be particularly pronounced among smaller businesses. In the current crisis, however, there is growing public concern that this process of creative destruction and ‘cleanse out’ of unviable firms is seriously hampered by an increasing policy-induced ‘zombification’ of the economy (see, e.g., The Economist 2020a; The Washington Post 2020). Analyzing only the short-term effects of policy aid on firm survival, we do not want to go as far as speaking of a zombification which typically refers to situations in which credit misallocation by banks sustains the survival of de facto insolvent firms over a longer period of time. Still, we hypothesize that the early policy measures with strong focus on SME relief induced an *insolvency gap*, defined as backlog of corporate insolvencies which are usually to be expected in a crisis like this.

If efficient resource reallocation and business liquidation are compromised through policy interventions, this has immediate consequences on entrepreneurship. Focusing on Germany, a country where liquidity support for SMEs has not only been particularly strong by international standards (Anderson & et al., 2020; OECD, 2020a) but also been accompanied by a temporary suspension of the obligation to file for insolvency (Federal Ministry of Justice and Consumer Protection, 2020), we identify the insolvency law as an important institutional determinant for entrepreneurship dynamics. Past literature has shown that (changes in) the institutional environment have an important influence on entrepreneurial outcomes (Baumol, 1990; Acs et al., 2008; Peng et al., 2010; Levie et al., 2014; Chowdhury et al., 2015; Arcuri & Levratto, 2020) determining both entrepreneurial exit but also firm entry (Melcarne & Ramello, 2020). Empirical results suggest that entrepreneur-friendly insolvency laws, characterized primarily by speed and efficiency in liquidation and reorganization processes, have a positive impact on new firm entry (Chemin, 2009; Lee et al., 2011). Moreover, research shows that the design of bankruptcy laws can favor high-growth entrepreneurship (Estrin et al., 2017; Eberhart et al., 2017). In fact, entrepreneurs seem to incorporate the efficiency of insolvency legislation into their founding decision as regions with faster liquidation proceedings appear to be associated with higher levels of business formations and firm growth (García-Posada & Mora-Sanguinetti, 2015; Melcarne & Ramello, 2020). However, entrepreneur-friendly insolvency laws can also have adverse impacts on start-ups and SMEs as refinancing costs may increase and access to credits is tightened by banks accordingly (Djankov et al., 2007; Berger et al., 2011; Rodano et al., 2016). On the exit side, literature points out that changes in the design of insolvency legislation strongly determine which type of firms predominantly initiate insolvency proceedings. In the late 1990s, for instance, various European countries have introduced formal restructuring procedures to allow reorganization of distressed firms (Brouwer, 2006). It appears, however, that the introduction of formal restructuring has barely been used by small firms as the costs of reorganization proceedings are often too high for smaller, financially constrained companies (Cook et al., 2001; Dewaelheyns & Van Hulle, 2008). Thus, for small entrepreneurial firms, insolvency declarations often offer no realistic path towards reorganization but are more likely to end in liquidation. Since the prospects of reorganization are low, insolvent small business owners have an additional incentive not to file for bankruptcy, which is why we argue that the temporary filing suspension is disproportionately used by smaller firms.

Besides Germany, further countries such as France, Spain, Luxembourg, Poland, Portugal, Russia and the Czech Republic have temporarily released corporate directors and entrepreneurs from their insolvency filing obligation in response to the pandemic. Other countries such as the USA temporarily raised the debt threshold for small businesses eligible to participate in reorganization proceedings (Gurrea-Martínez, 2020). Using insolvency data on French firms, Cros et al. (2021) find that insolvency rates have substantially fallen below pre-crisis rates. However, they argue that the selection process to file for insolvency has not been distorted during the pandemic because firm characteristics that determine failure and survival have remained unchanged compared to pre-crisis times. For the US economy, Wang et al. (2020) find a sharp decline in insolvency filings among small firms, while bankruptcy proceedings among large firms remain at normal levels. Despite the eased access to reorganization for smaller firms, they suggest that small businesses see insolvency proceedings only as a last resort because successful reorganization is unlikely and often too costly. In general, official figures show that corporate insolvency numbers after the outbreak of the crisis have strongly decreased compared to 2019 levels especially in countries which implemented changes in their insolvency frameworks (see Fig. 7 in the Appendix). This underpins the idea that the large-scale governmental support programs have, indeed, led to substantial distortions in business dynamics. Clearly, the suspension has allowed entrepreneurs with viable business models to stay in the market and use public liquidity subsidies to avert insolvency. But at the same time, if unprofitable firms do not exit the market because they are not required to do so, the efficient reallocation of resources is impeded. Access to skilled human capital, physical resources such as office space, and bank loans is limited for newly entering firms when unviable firms congest the market. This may prevent future entrepreneurs from starting up, but can also discourage existing entrepreneurs from initiating new, more promising ventures. Hence, we assume that an insolvency gap along with a further prolongation of aid measures in the ongoing crises is likely to result in a decrease in entrepreneurial activity in the longer term.

## Policy response in Germany

Official figures show that in Germany, the fiscal policy response to prevent corporate insolvencies due to crisis-related liquidity bottlenecks is particularly pronounced by international comparison. According to a comparative study of the economic think tank Bruegel, nearly 40% of Germany’s 2019 GDP was spent on COVID-19 measures to strengthen companies’ liquidity positions (Anderson et al., 2020). Compared with a number of selected OECD countries, this is the second strongest response in terms of a country’s overall economic performance (see Fig. 6 in the Appendix). In fact, the German Federal Government itself describes the response as the ‘largest assistance package in the history of the Federal Republic of Germany’ (Federal Ministry of Finance 2020d, 3).

From a small business economics view, it is interesting to see that a number of intervention measures adopted by the German Federal Government have been specifically designed to target SMEs (OECD, 2020a). In the following, we describe the policy instruments to counter the economic impacts of the COVID-19 crisis in more detail, focusing on how the instruments differ with respect to firm size (for a quick overview of the measures and possible effects on corporate insolvencies the reader is referred to Table 10 in the Appendix).

### Direct liquidity subsidies

As an immediate response to the first lockdown, the Federal Government granted liquidity subsidies through direct cash transfers (‘Sofort-’ and ‘Überbrückungshilfen’). The extent of liquidity support is primarily determined by company size, measured by the number of employees or previous revenues. In case of the ‘Soforthilfen’, for instance, only micro-firms with up to 10 employees were eligible to receive injections between €9,000 and €15,000 for three months to cover their operational costs (Federal Ministry of Finance, 2020d). These immediate subsidies have been accompanied by a large-scale stimulus package worth €25 billion covering a substantial part of SMEs’ fixed operating costs (Federal Ministry of Finance, 2020c). Generally, the subsidies were granted in a non-bureaucratic fashion easily accessible to all micro-businesses and SMEs which assured that they were suffering financial distress because of the COVID-19 pandemic (Federal Government of Germany, 2020).

### Liquidity loans under public guarantee schemes

For SMEs with more than 10 employees the KfW Instant Loan Program has been launched. The program offers SMEs loans that are fully collateralized by the state. These loans amount up to 25% of a firm’s 2019 revenues with a cap of €500k for small companies and €800k for medium-sized companies, respectively. No credit risk assessments are taking place and no collaterals are required. The only eligibility criterion is that the company was profitable in 2019 or at least on average profitable between 2017 and 2019 (Federal Ministry for Economic Affairs and Energy, 2020). This fairly broad criterion shows that the process is focused on speed and ease applied ‘without red tape’ (Federal Ministry of Finance 2020b, 1) and not on elaborate screening mechanisms that could prevent providing liquidity to unviable firms.

Furthermore, the COVID-19 support package includes additional government guarantees on loans for both small and larger businesses, including lower interest rates for small firms compared to large firms. (Federal Ministry for Economic Affairs and Energy, 2020). Similar to the Instant Loan Program, the loans are channeled through commercial banks and the state-owned bank KfW assumes risk coverage of 80% for large enterprises and 90% for SMEs with a simplified risk assessment (Federal Ministry of Finance, 2020a). For commercial banks, this makes lending to SMEs particularly attractive and, given that they only bear 10% of the default risk, further disincentivizes comprehensive risk assessments by the issuing bank.

### Liquidity support through labor cost subsidies

Another form of liquidity support to companies is the use of short-time compensations (‘Kurzarbeitergeld’) which are direct subsidies on firms’ labor costs. This instrument has been available for quite some time; however, its eligibility criteria were relaxed in the pandemic. Now companies with only 10% of employees working on short-time qualify for the wage subsidy (instead of one third) (OECD, 2020b). In addition, the subsidy has been increased compared to pre-crisis levels, ranging now from 60 to 87% of the worker’s last net income. From a company perspective, short-time compensations reduce labor costs, allow the company to retain specific human capital and avoid the costs of new hires and training when the economy recovers again. Drawing on literature from the Great Recession, the usage of short-time work (STW) has a positive impact on firm survival (Cahuc et al., 2018; Kopp & Siegenthaler, 2021) but at the same time research results suggest that low productivity firms have been taken up STW more often (Giupponi & Landais, 2018). From a welfare perspective, this may have adverse effects as it impedes the reallocation of workers from low- to high-productivity firms. Since SMEs tend to be active in more labor-intense business activities than larger firms (Yang & Chen, 2009), it is reasonable to assume that SMEs as well as labor-intense sectors benefit disproportionately from short-time compensations. Eligibility criteria for STW are unrelated to firms’ pre-crisis performance, which allows unviable companies to benefit from the instrument as well.

### Intertemporal liquidity support

To further improve the liquidity situation of companies, authorities have granted tax payment deferrals, allowed lower tax prepayments and suspended enforcement measures for tax debts. The tax-related intertemporal liquidity assistance amounts to an estimated €250 billion and the policy measure applies equally to all company size classes (Anderson & et al., 2020).

### Temporary change in insolvency law

Finally, the different elements of liquidity provision which have been granted to German businesses were accompanied by a temporary amendment to the German insolvency law. On March 27, 2020, the Federal Government decided to temporarily suspend the insolvency filing obligation in order to avoid a massive increase in insolvencies as a result of COVID-19-induced liquidity shortages. The obligation to file an insolvency has been suspended until September 30, 2020, with an adjusted extension until the beginning of 2021. Although the amended law stipulates that only those firms that are insolvent or over-indebted due to the COVID-19 pandemic are temporarily exempt from insolvency proceedings, policy makers face the dilemma that it is barely possible to assess whether insolvent *non-filers* fulfill this eligibility criterion. This is particularly true for smaller firms, whose limited disclosure requirements make such an assessment even harder. While there is no doubt that many viable companies facing illiquidity and over-indebtedness as a result of the economic shock will benefit from the law change, it also creates loopholes for smaller, unviable companies to stay in the market and absorb public liquidity aid.

The two cornerstones of the aid measures—public liquidity support and the amendment of the insolvency law—have been implemented simultaneously as a joint strategy to prevent widespread corporate insolvencies. Therefore, we cannot differentiate which influence the individual measures have on the emergence of a possible insolvency gap. However, we argue that the policy response must be understood as a mix of interdependent policy actions that likely would not have been effective in preventing business failures had they been implemented separately. In particular, the liquidity provision through state-supported loans and the temporary suspension of the filing obligation have only had an insolvency-preventing effect because they were implemented simultaneously and mutually. Without the filing suspension, companies would have been discouraged from taking out government loans as this would have led many of them into over-indebtedness, which in normal times would have obliged firms to declare insolvency. Likewise, without liquidity provision through easily accessible loans and other subsidies, the sole insolvency suspension would have been ineffective since in light of strongly diminished turnovers the economic reality of many liquidity constrained firms would have implied a de facto insolvency. Following this line of reasoning, the effect of liquidity support and temporary change in insolvency law on (non-) selection into insolvency is best analyzed as a policy mix used to combat the threat of mass insolvencies. While the insolvency filing suspension allowed both small and large companies to avert insolvency and possibly survive the crisis by taking advantage of liquidity injections by the state (Federal Ministry of Justice and Consumer Protection, 2020), it has been shown that many of the liquidity support measures directly target SMEs or provide indirect channels for especially smaller (and often entrepreneurial) businesses to benefit disproportionately. With few screening mechanisms in place, there is the risk that unviable firms will be kept alive, freezing up resources that could be used more productively elsewhere and possibly hampering entrepreneurial activity.

This section has highlighted the role of policy support to counter the economic consequences of the pandemic in Germany—a country that has provided substantial assistance to businesses to avoid a wave of corporate bankruptcies. It has suggested that the joint implementation of widespread but undifferentiated liquidity support strongly focusing on SMEs together with the temporary amendment of the insolvency law, is likely to have favored a backlog of corporate insolvencies particularly pronounced among small and possibly financially weak companies. In the next section, we introduce the data and methodology we use to estimate the existence and extent of such an insolvency gap.

## Data, variables and methodology

### Data and variables

The study uses two data sources which both originate from the Mannheim Enterprise Panel (MUP) covering the near universe of economically active firms in Germany (Bersch et al., 2014). The first data source is a survey where the questioned companies have been sampled from the MUP. The survey is used to examine how companies of different size and in different sectors are affected by the adverse impacts of the COVID-19 pandemic and motivates why we estimate the insolvency gap distinguishing between sector affiliation and company size. For the estimation of the insolvency gap we use a second data source: a large sample of firm-specific credit rating information along with information concerning the firms’ insolvency status. In the following, we will introduce both data sources and the variables used in this study in more detail.

#### Survey data

**Fig. 1:**
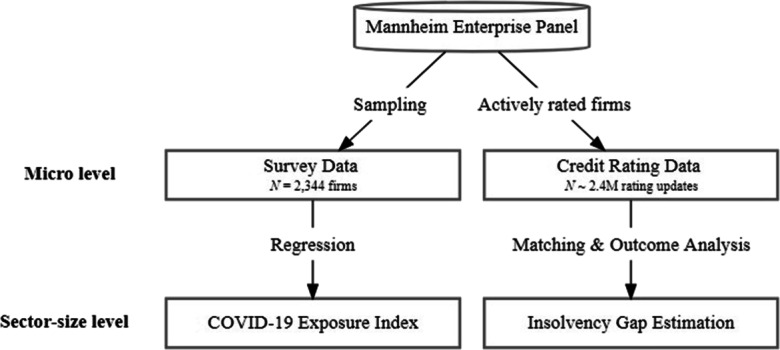
Data sources used in this study. Note: Observations of the survey data (companies) and credit rating data (firm-specific rating revisions) originate from the Mannheim Enterprise Panel (MUP) data base. Survey data allows to estimate exposure to adverse effects of the pandemic on the sector-size level. Credit rating data is used to estimate the existence of an insolvency gap on the sector-size level

We employ the survey to primarily assess which industries and company sizes are affected most by the crisis. Based on a representative random sample of German companies, drawn from the MUP and stratified by firm size and industry affiliation the survey was conducted three times spanning the period in which the German insolvency regime was fully suspended.^2^ The survey includes questions on COVID-19-related economic effects on various business dimensions. The collected data has then been supplemented with credit rating scores from the MUP, which allows to control for the financial situation of the companies prior to the crisis. As shown in Fig. 1, we use the survey data to investigate whether the adverse economic impacts of COVID-19 differ across sectors and firm size classes. These results together with the heterogeneity in public aid programs with respect to firm size as outlined in Section 3 motivates us to conduct our main empirical analysis at the sector-size level.

Table 1 shows summary statistics of the relevant variables used to construct a COVID-19 Exposure Index, *CEI*, reflecting the extent to which firms experienced negative impacts in relation to the pandemic. Firms were asked on a Lickert scale of 0 to 4 in which of the following areas they experienced negative impacts as a result of the COVID-19 crisis: (1) decrease in demand, (2) shutdown of production, (3) supply chain interruption, (4) staffing shortage, (5) logistical difficulties, (6) liquidity shortfalls.^3^ From these six questions we construct *CEI* as simple sum of the response values. The average index is 6.31 out of a maximum possible value of 24. The most common and most severe impact relates to the decline in demand, where respondents reported an average negative impact of 1.85. Shutdown of production facilities and liquidity bottlenecks are also frequently mentioned consequences.

#### Credit rating data

For the purpose of estimating whether the bankruptcy filing behavior has changed significantly as a result of the crisis-related aid measures and possibly created a backlog of insolvencies, we examine credit rating updates of close to all economically active firms listed in the MUP.^4^ The Mannheim Enterprise Panel is particularly suited for an analysis of the insolvency-related cleansing effect as it is constructed by processing and structuring data collected by Creditreform, the leading credit agency in Germany. Creditreform regularly measures and updates the creditworthiness of German companies. Overall our sample comprises 2,373,782 credit rating updates of 1,500,764 distinct German businesses whose ratings were updated at least once during the last three years.^5^ Table 2 shows that the sample of about 1.5 million companies is very diverse in its industry and size composition. Most important in the context of this study is the coverage of SMEs, which not only is representative for the German economy (see Table 2), but also allows for a nuanced differentiation between medium-sized, small and micro-enterprises. Therefore, it suits well to examine the policy-induced heterogeneity of the COVID-19 related effects on business failures with a special focus on possible size differences not only among SMEs and large enterprises but also within the group of SMEs. The latter estimation of the insolvency gap will be conducted on the sector-size level as displayed in Table 2.

Assuming that the COVID-19 shock and its economic consequences on liquidity and insolvency distress of German businesses began by the end of March 2020, we split our sample into a ‘pre-crisis’ period and a ‘crisis’ period. This cutoff point also captures COVID-19 policy dynamics as the German government imposed the first countrywide lockdown that includes a shutdown of most customer service-related businesses on March 22 and suspended the obligation to file for bankruptcy on March 27 (Federal Ministry of Justice and Consumer Protection, 2020). Consequently, the pre-crisis period comprises all credit rating updates which took place between July 2017 and December 2019. The crisis period includes all observations between April 2020 and end of July 2020.^6^ In the later estimation of the insolvency gap, rating updates from the pre-crisis period serve as pool of control observations. Closely matching credit rating updates from this pool are used to estimate counterfactual insolvency rates which will be compared against the actual insolvency rates observed after April 1, 2020.^7^

**Fig. 2:**
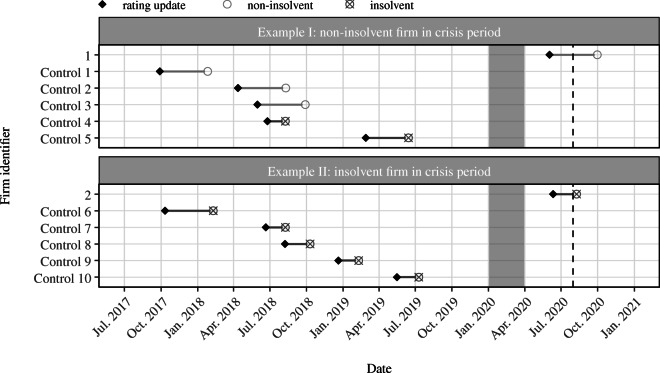
Matching: illustration. Note: The figure illustrates the nearest neighbor matching for two micro-enterprises in the accommodation and catering sector. In the top panel, we see that firm 1 experienced a rating update in the crisis period which did not result in an insolvency filing. Furthermore, we see, however, that two out of the *k* = 5 nearest neighbors from the pre-crisis control period filed for insolvency after they received a very similar rating update. This signals that firm 1, given its financial information, faces a relatively high insolvency risk as almost half of its nearest neighbors indeed went bankrupt in times without policy intervention. The bottom panel shows the same approach but for firm 2 which filed for insolvency shortly after its rating update during the crisis period. We see that all of the nearest neighbors also filed for insolvency and thus closely reflect the actual survival status of firm 2. If we do not observe an insolvency filing four months after the rating update, we treat the update as non-insolvent. Therefore, the time between rating update and the non-insolvent labelling in the visualization always spans 4 months. The area shaded in gray highlights a transitional phase which we intentionally exclude from our analysis since assignment of observations falling in that phase to either the pre-crisis or the crisis period is not straightforward. The dashed vertical line at the end of July 2020 signals that we only have credit rating updates available up to this point. Note, however, that we observe insolvency filings beyond this point in time

**Table 1:** Descriptive statistics: survey data

Variables	*N*	Mean	SD	Min	Max
COVID-19 Exposure Index (*CEI*)	2,344	6.31	5.40	0	24
Questions used for the index calculation					
(1) Decrease in demand	2,344	1.85	1.56	0	4
(2) Lockdown of production	2,344	1.05	1.58	0	4
(3) Supply chain interrupted	2,344	0.88	1.24	0	4
(4) Staffing shortage	2,344	0.64	1.04	0	4
(5) Logistical difficulties	2,344	0.81	1.28	0	4
(6) Liquidity shortfalls	2,344	1.08	1.41	0	4

Note: The table shows descriptive statistics of the COVID-19 Exposure Index (*CEI*). It also displays statistics of the survey questions used to construct the index

**Table 2:** Sample decomposition of credit rating data

Sector affiliation	Size of company				Total
	Micro	Small	Medium	Large	(sample)
Business-related services	89.4%	8.3%	1.9%	0.4%	28.6%
Manufacturing	84.9%	11.8%	2.7%	0.6%	22.5%
Wholesale & retail trade	83.1%	13.4%	2.9%	0.6%	19.9%
Health & social services	84.8%	10.6%	3.5%	1.1%	7.3%
Insurance & banking	93.6%	3.6%	1.8%	1.0%	4.5%
Accommodation & catering	88.5%	9.8%	1.6%	0.1%	4.1%
Logistics & transport	80.5%	15.3%	3.5%	0.7%	4.1%
Others	82.7%	10.2%	4.6%	2.5%	3.9%
Creative industry & entertainment	88.9%	8.8%	2.0%	0.3%	1.6%
Mechanical engineering	54.3%	27.5%	13.0%	5.2%	1.3%
Food production	64.3%	23.0%	10.3%	2.4%	1.0%
Chemicals & pharmaceuticals	49.1%	29.1%	16.5%	5.3%	0.7%
Manufacturing of data processing equipment	58.9%	26.7%	10.9%	3.5%	0.5%
Total (sample)	85.2%	11.1%	2.9%	0.8%	100%
Total (population)^a^	81.8%	15.1%	2.5%	0.6%	100%

Note: The table shows the company size distribution within sectors (rows) as well as the sector distribution (column ‘Total (sample)’) in our credit rating sample. Size classification is determined by number of employees, annual turnover and annual balance sheet total following the recommendation of the EU Commission (European Commission, 2003) as outlined in Table 8 in the Appendix. Sector groups are built to reflect anecdotal heterogeneity in the context of COVID-19. Grouping of sectors is based on EU’s NACE Revision 2 classification scheme (European Union, 2006). In Table 9 in the Appendix an exact mapping of sector groups and NACE divisions can be found. In all sectors the fraction of SMEs lies far above 90% which makes the data particularly useful to analyze the effects of COVID-19-related policy responses on smaller firms. Also note that the overall size composition of our sample compares well against the official size distribution of the population of German active companies as reported by the Federal Statistical Office (Destatis, 2020)

^a^ Population size distribution according to official statistics of the Federal Statistical Office (Destatis, 2020)

For the estimation of insolvency rates, we enrich our sample of firm-specific credit rating data with information on the firm’s survival status after it has received an update on its rating. Information on firm-specific survival states is obtained by the online register for bankruptcy filings of the German Ministry of Justice. Besides information identifying the companies which have filed for insolvency, the register also contains the filing date, allowing us to match the most recent rating update that predates the filing date for that particular bankrupt firm. Our overall sample comprises 15,634 credit rating updates that were followed by an insolvency and 2,358,148 rating updates which did not result in an insolvency filing. With this data, we are able to estimate two statistics. First, we use this information to estimate bankruptcy rates after the COVID-19 outbreak on the sector-size level based on firms for which we observe credit rating updates during the pandemic. Second, using comparable firms with closely matching credit rating updates in non-crises times as control group, we are able to estimate counterfactual insolvency rates. Comparing observed insolvency rates with counterfactual insolvency rates within each of the sector-size strata allows us to obtain sector-size-specific estimates of the insolvency gap. In addition to firm size, industry affiliation, and credit rating update, we consider an extensive set of additional firm-specific variables when matching counterfactual survival states of pre-crisis observations with rating updates of firms observed in the COVID-19 period. In the following section, we introduce all of these matching variables and provide some descriptive statistics.

In our data used for the estimation of the insolvency gap firm survival status, *f*_*t*+ 4_, serves as outcome variable. It is equal to 1 if the company has filed for insolvency no more than four months after its last rating update. If the firm has not gone bankrupt or it has filed insolvency more than four months after its latest rating update, it is 0. This means that we take four months as maximum time lag between a credit rating update and the date at which the respective firm has filed its bankruptcy to count the rating update as being predictive for the subsequent insolvency filing. We choose this threshold for two reasons. First, we want to ensure that the rating update has a high information content in predicting a potential insolvency filing. If the date of bankruptcy lies more than 4 months after the credit update, it is likely that the update does not reflect the reasons why the company went bankrupt. A more recent update of the firm’s rating (if that existed) would be necessary to capture the company’s financial deterioration that contributed to the subsequent insolvency. Second, the COVID-19 period for which we have information on credit rating updates spans 4 months from April 2020 to the end of July 2020. Thus, for the latest in-crisis rating updates in July 2020, we can observe the firm’s survival status at most 4 months until November 2020 (the time of writing this paper). Therefore, the maximum forecasting horizon for the rating updates observed in the crisis period is limited to 4 months.

The most important variable in finding counterfactual survival states in the matching procedure is Creditreform’s credit rating index since it is the basis for the calculation of the credit rating updates. The credit rating is calculated by Creditreform on the basis of a rich information set relevant to assess a company’s creditworthiness. The metrics considered in calculating the rating include, among other things, information on the firm’s payment discipline, its legal form, credit evaluations of banks, credit line limits and risk indicators based on the firm’s financial accounts (if applicable) (Creditreform, 2020b). Creditreform attaches different weights to these metrics according to their relevance on determining a firm’s risk of credit default and calculates an overall credit rating score which ranges from 100 to 500.^8^ The higher the score, the worse the firm’s creditworthiness and thus the higher the risk of insolvency. In fact, Creditreform’s rating index has a high forecasting quality to assess a firm’s credit default risk (Creditreform, 2020b). Assuming that a high credit default risk signals financial distress, which often results in insolvency, we use Creditreform’s rating as the basis for predicting corporate insolvency risk. The prediction of corporate bankruptcy via a scoring model goes back to the seminal work of Altman (1968) and his development of the Z-score model. Similar to Creditreform’s credit rating index, the Z-score model relies on several accounting-based indicators which are weighted and summed to obtain an overall score. This score then forms the basis for classifying companies as insolvent or non-insolvent (Altman, 2013). Today, this model approach is still used by many practitioners to predict firm insolvencies (Agarwal and Taffler, 2008).

Based on the credit rating index, we construct the following variables. Our main predictor variable is the *update* in the rating index, Δ*r*_*t*_, which is defined as the difference of the new rating assigned by Creditreform an the rating before the update (Δ*r*_*t*_ = *r*_*t*_ − *r*_*t*−*x*_).^9^ Given the logic of the rating index, a positive sign in the rating update reflects a downgrade in financial solvency, a negative sign reflects an improvement in the rating, i.e. an upgrade of the company’s financial standing. The amount of the down-/upgrade reflects how severely the company’s financial standing has changed.^10^

Apart from the rating update, we also consider the rating before the upgrade, *r*_*t*−*x*_, as a matching variable when predicting counterfactual insolvency states. This allows us to control for the location of the company in the rating distribution and consequently how high the default risk was before the down-/upgrade. Moreover, we form two additional variables from the firm’s credit rating information, both of which control for the medium-term path of the firm’s financial standing. First, we count the number of downgrades in the three years preceding the update at hand, *d*_*t*_. Second, we calculate the average credit rating in the three years prior to the current update under consideration, $\bar {r}_{t}$.^11^ Finally, we consider firm age, *a*_*t*_, as further matching variable acknowledging that younger firms tend to be more prone to insolvency.

Table 3 shows descriptive statistics of the variables considered in the matching procedure. We see that an update which is followed by a bankruptcy filing relates to a downgrade of close to 70 scoring points on average. This is a substantial deterioration in the rating index compared to an update which is not followed by an insolvency filing. In fact, the difference in means between non-insolvency-related updates and insolvency-related updates, as reported in column ‘Δ Mean’, amounts to more than 65 index points and is statistically significant. For all other matching variables, we also find statistically meaningful differences suggesting that firms which go bankrupt have a worse credit rating both short-term and mid-term, have experienced more downgrades in the past and are younger on average. The economically and statistically significant differences between non-insolvency-related and insolvency-related credit updates across all variables suggest that they serve well as matching variables in a counterfactual estimation of insolvency rates.

**Table 3:** Descriptive statistics: non-insolvent observations and insolvent observations

Variable	Non-insolvent					Insolvent					Δ Mean
	*N*	*N* firms	Min	Mean	Max	*N*	*N* firms	Min	Mean	Max	
Predictor variables											
Credit rating update: Δ*r*_*t*_	2,358,148	1,489,376	− 356	4.0099	351	15,634	15,634	− 226	69.6892	359	− 65.6793***
Credit rating (prior to update): *r*_*t*−*x*_	2,358,148	1,489,376	100	266.5879	500	15,634	15,634	141	414.6607	500	− 148.0728***
Number of downgrades (3-year horizon): *d*_*t*_	2,358,148	1,489,376	0	0.4797	3	15,634	15,634	0	0.5051	3	− 0.0254***
Average credit rating (3-year horizon): $\bar {r}_{t}$	2,358,148	1,489,376	100	265.8216	500	15,634	15,634	138	367.6691	500	− 101.8475***
Company age: *a*_*t*_	2,346,686	1,479,383	1	22.2604	1,017	15,166	15,166	1	13.3199	400	8.9405***

Note: Non-insolvent observations comprise credit rating updates which have not resulted in an insolvency filing in the first four months after the update. Insolvent observations include observations which have been followed by an insolvency filing in the first four months after filing. *N* refers to the number of rating updates, *Nfirm* to the number of unique firms which experienced at least one rating update. Significance levels: *p < 0.10, **p < 0.05, ***p < 0.01

We also report univariate descriptive statistics of our credit rating sample differentiating between the pre-crisis and the crisis period in Table 4. We see that before the COVID-19 outbreak 0.71% of rating updates were followed by a bankruptcy filing. This translates into an insolvency filing rate of 1.05% on the firm level (note that firms can receive more than one credit rating update in that period). In the crisis period, however, despite the worsened economic conditions, it turns out that only 0.33% of rating updates were followed by a bankruptcy filing. This fraction also equals the firm-level insolvency filing rate as in the 4-month crisis period each firm is only observed once. ‘Δ Mean’ reporting the difference between the variable means of the pre-crisis and the crisis period suggests that the difference of 0.38 pp in the average survival status is statistically significant. The lower average insolvency rate in the crisis period contrasts with the finding that the financial rating of firms observed in the crisis period has deteriorated on average. In fact, firms experience, on average, a significantly higher downgrade of more than three index points during the crisis period.^12^ This decline of insolvencies in the COVID-19 period is consistent with official figures (The Economist, 2020b) and is a first indication that there is indeed an insolvency gap in the German economy. The strong political reaction to strengthen firms’ liquidity and to prevent German companies from going bankrupt is likely to be a driving force behind the low insolvency rate in the crisis period.

**Table 4:** Descriptive statistics: pre-crisis observations and crisis observations

Variable	Pre-crisis					Crisis					Δ Mean
	*N*	*N* firms	Min	Mean	Max	*N*	*N* firms	Min	Mean	Max	
Outcome variable											
Survival status: *f*_*t*+ 4_	2,036,103	1,377,671	0	0.0071	1	337,679	337,679	0	0.0033	1	0.0038***
Predictor variables											
Credit rating update: Δ*r*_*t*_	2,036,103	1,377,671	− 356	3.9825	359	337,679	337,679	− 293	7.2161	349	− 3.2336***
Credit rating (prior to update): *r*_*t*−*x*_	2,036,103	1,377,671	100	267.5344	500	337,679	337,679	100	267.7361	500	− 0.2017**
Number of downgrades (3-year horizon): *d*_*t*_	2,036,103	1,377,671	0	0.4812	3	337,679	337,679	0	0.4714	3	0.0098***
Average credit rating (3-year horizon): $\bar {r}_{t}$	2,036,103	1,377,671	100	266.3589	500	337,679	337,679	100	267.2973	500	− 0.9384***
Company age: *a*_*t*_	2,024,173	1,367,244	1	22.0970	1,017	337,679	337,679	1	22.8378	1,016.00	− 0.7408***

Note: Pre-crisis period comprises all credit rating observations from July 2017 to December 2019. Crisis period includes all observations starting from April 2020 to July 2020. Although the mean differences in the predictor variables are statistically significant, their magnitude seems to be rather negligible (except credit rating update), particularly when comparing with the differences between non-insolvent and insolvent observations (Table 3). This suggests that the crisis sample is not biased in the sense that it primarily includes credit updates of firms with poor financial records. Significance levels: *p < 0.10, **p < 0.05, ***p < 0.01

It remains to be analyzed if there are specific sector-size combinations for which the number of insolvencies is significantly below the counterfactual number that one would expect given the observed rating updates and information from pre-crisis insolvency paths. Also we aim to tackle the question whether the gap is mainly driven by firms which already before the crisis were characterized by a weak financial standing. In the next section, we introduce a matching approach that allows us to predict counterfactual insolvency filings based on pre-crisis observations where no policy intervention saved struggling firms from insolvency. With this approach, we are able to derive counterfactual insolvency rates at the sector-size level and provide an estimate regarding the existence of an insolvency gap by comparing them with the actual filings observed during the crisis period.

### Methodology

#### Nearest neighbor matching

This paper focuses on the extent to which government policy in the COVID-19 crisis may have induced ailing firms to stay in the market. To answer this question, we compare the survival status of closely matching firms observed before the COVID-19 outbreak with the survival status of firms observed during the pandemic. Besides general company characteristics such as company size, industry affiliation and company age, our matching approach takes particular account of firm-specific solvency information as presented in the previous section. The core idea of the matching procedure is to find comparable firms which have experienced very similar rating updates and have followed an almost identical path in their financial solvency but in times prior to COVID-19 and the related policy interventions that keep struggling firms afloat.

We conduct a nearest neighbor matching approach in order to find for each of the in-crisis observations a number of matches from the pre-crisis period. Nearest neighbor matching in observational studies goes back to the work of Donald Rubin (1973) and aims at reducing bias in the estimation of the sector-size-specific insolvency gap. A simple comparison of the mean values of the survival status of observations before the crisis and during the crisis (as in Table 4) is likely to give a highly biased picture of the insolvency gap. First, policy measures to rescue firms from failing have been highly heterogeneous with respect to firm size as highlighted in Section 3. Therefore, comparing the survival status of firms of different size bears high risk of firm size acting as confounding variable in the estimation of a policy-induced backlog of insolvencies. For this reason, we only search for matches within the same company size group. Next, the evaluation of our survey suggests that there is great heterogeneity in the COVID-19 exposure across sectors (as becomes apparent in Section 5.1). For this reason, we only match firms that are in the same sector class. Ultimately, the previous section has shown that in the crisis period the distribution of rating updates has systematically shifted to the right implying that the in-crisis observations have, on average, experienced larger downgrades in their ratings. For an unbiased estimation of the insolvency gap, this shift needs to be controlled for. Our nearest neighbor matching aligns the in-crisis distribution of updates with the distribution of matched observations as we put a strict caliper on the credit rating variable when searching for matching observations. In fact, comparing the distribution of the predictor variables between pre-crisis and crisis period before and after matching indicates that control observations and crisis observations are much more balanced after matching (see Table 12 in the Appendix for an assessment of covariate balance).

The details of our matching algorithm look as follows. Acknowledging the heterogeneity with respect to firm size and sector affiliation, we estimate the insolvency gap within each of the 52 sector-size combinations. Therefore, we only consider pre-crisis observations that share the same sector-size stratum as the crisis observation of interest. In that sense we perform exact matching on both sector affiliation and company size group. Next, within each sector-size stratum the algorithm selects for each in-crisis observation *i* the *k* nearest neighbors from the pre-crisis period which have the smallest distance from *i*. The maximum number of nearest neighbors, *k*, reflects the ratio of pre-crisis and crisis observations within each sector-size stratum. Distance is measured by the Mahalanobis distance metric (Rubin, 1980), *MD*, which is computed on all predictor variables $\boldsymbol {X} = ({\Delta } r_{t} r_{t-x} d_{t} \bar {r}_{t} a_{t})'$. For the key predictor variable, Δ*r*_*t*_, we additionally impose a caliper, *c*, of 0.25 standard deviations. Thus, a pre-crisis observation, *j*, only falls under the *k* nearest neighbors if it does not exceed the caliper on Δ*r*_*t*_.

$$ \begin{array}{@{}rcl@{}} MD_{ij} = \left\{\!\! \begin{array}{ll} (\boldsymbol{X}_{i} \! - \! \boldsymbol{X}_{j})^{\prime}\!\boldsymbol{\Sigma}^{ - 1}(\boldsymbol{X}_{i} \! - \! \boldsymbol{X}_{j}) & \text{if} \quad\!\!\! \vert {\Delta} r_{t, i} - {\Delta} r_{t, j} \vert \!\leq \!c\\ \infty & \text{if} \quad \!\!\!\vert {\Delta} r_{t, i} - {\Delta} r_{t, j} \vert \!>\! c \end{array} \right. \end{array} $$

with **Σ** as the variance covariance matrix of ***X*** in the pooled sample of in-crisis and all pre-crisis observations. The strict caliper implies that the number of matches on each crisis observation can be smaller than *k* or, in case that there is no control observation fulfilling the caliper condition, there may even be no match. If this the case, the crisis observation for which no match could be found is disregarded from further analysis. Moreover, we conduct matching with replacement allowing pre-crisis units to match to more than one crisis observation. In the outcome analysis, this requires us to consider weights which reflect whether a pre-crisis unit falls in the matched sample more than once. In Section 5.2 where we estimate the insolvency gap on the sector-size level, we need to consider these weights for inference (Stuart, 2010). In this way, we can not only predict the crisis observations’ probability to file for bankruptcy if there was no policy intervention but also make a statement whether the differences between the observed insolvency rates and the predicted counterfactual insolvency rates on the sector-size level are statistically significant.

Before presenting the results of the counterfactual insolvency rate prediction and insolvency gap estimation, we use our survey results in the next section to show how the pandemic affected sectors to varying degrees. The observed heterogeneity in sector exposure motivates our further empirical analysis.

## Empirical results

### COVID-19 exposure and firm characteristics

Anecdotal evidence suggests that industries are asymmetrically affected by the COVID-19 recession because lockdown measures as well as supply and demand effects differed between sectors. To verify this observation, we empirically investigate to what extent the economic effects of the COVID-19 crisis have asymmetrically hit sectors by making use of our survey data. In addition, we analyze whether firm size and the pre-crisis credit rating is correlated with the perceived shock by the COVID-19 recession at the firm level.

The regression results of the analyses are shown in Table 5. Model (1) reveals that the COVID-19 Exposure Index indeed significantly differs between sectors. We choose chemicals and pharmaceuticals as reference category since this sector is least negatively affected. The sectors accommodation and catering as well as creative industry and entertainment experience very strong and significant negative shocks in comparison to the baseline sector. This is in line with the strong restrictions experienced in these sectors. Since the business activities in these sectors often require direct human interactions, corresponding companies have been severely affected by lockdown measures. Interestingly, firm size categories show no statistically significant heterogeneity in their correlation with the COVID-19 Exposure Index as Model (2) reveals. The effects with respect to sectors and firm size also hold when both measures are incorporated simultaneously as in Model (3). Controlling further on the firms’ pre-crisis credit rating and thus on the financial situation prior to the outbreak shows that the rating is significantly correlated with the perceived COVID-19 impact. Although the effect is low in magnitude, the marginal effect suggests that a higher (worse) credit rating is associated with a stronger exposure to the negative impact of the crisis. Ultimately, the strong heterogeneity in the negative exposure to the economic consequences of the pandemic with respect to sector affiliation hold when controlling for the firms’ pre-crisis credit rating in Model (4).

**Table 5:** Regression: COVID-19 Exposure Index on firm characteristics

	(1)	(2)	(3)	(4)
	*CEI*	*CEI*	*CEI*	*CEI*
Business-related services	0.637		0.646	0.473
	(0.611)		(0.609)	(0.615)
Manufacturing	− 0.004		− 0.023	− 0.073
	(0.605)		(0.603)	(0.604)
Wholesale & retail	1.479**		1.476**	1.427**
	(0.647)		(0.644)	(0.646)
Health & social services	1.087*		1.085*	0.855
	(0.660)		(0.657)	(0.661)
Insurance & banking	0.643		0.618	0.653
	(0.689)		(0.686)	(0.682)
Acc. & catering	6.024***		6.046***	5.835***
	(0.711)		(0.710)	(0.712)
Logistics & transport	1.454**		1.464**	1.396**
	(0.650)		(0.647)	(0.646)
Creative i. & entertainment	5.444***		5.445***	5.224***
	(0.832)		(0.831)	(0.831)
Mechanical engineering	2.464***		2.477***	2.433***
	(0.665)		(0.659)	(0.658)
Food production	2.564***		2.559***	2.394***
	(0.701)		(0.699)	(0.696)
Manufac. of data proc. equip.	0.147		0.156	0.208
	(0.653)		(0.652)	(0.650)
Micro-enterprise		0.311	− 0.048	− 0.509
		(0.423)	(0.418)	(0.455)
Small enterprise		0.269	− 0.248	− 0.538
		(0.447)	(0.433)	(0.448)
Medium-sized enterprise		− 0.0216	− 0.128	− 0.209
		(0.457)	(0.440)	(0.444)
Credit rating (pre-crisis)				0.008***
				(0.003)
*N*	2,344	2,344	2,344	2,344

Note: Chemicals and pharmaceuticals serve as baseline sector among the sector dummies, large enterprises serve as baseline size group. Dummy coefficient estimates need to be read relative to the baseline group(s). Standard errors are reported in parentheses. Significance levels: *p < 0.10, **p < 0.05, ***p < 0.01

The heterogeneous COVID-19 exposure at the sector level shows that differences in insolvency dynamics with respect to industry affiliation may play an important role. Taking further into consideration that many of the policy measures in Germany have been specifically tailored to SMEs, the subsequent estimation of the insolvency gap is conducted at the sector-size level.

### The COVID-19 insolvency gap

#### Results on the sector-size level

Estimating the insolvency gap requires us to derive two statistics. First, we calculate actual insolvency rates, $IR^{actual}_{s}$, observed after the COVID-19 outbreak for each sector-size stratum *s*.^13^ The calculation is based on firms for which we observe credit rating updates after April 1, 2020.

$$ \begin{array}{@{}rcl@{}} IR^{actual}_{s} = \frac{N^{insolvent}_{s}}{N_{s}} \end{array} $$

Second, taking the matched sample of observations from the pre-crisis period which includes for each firm observed in the crisis period at most *k* nearest neighbors, we are able to estimate counterfactual insolvency rates, $IR^{counterfactual}_{s}$, as follows


with $\tilde {N}_{s} = {\sum }_{j=1}^{\tilde {N}_{s}} w_{j, s}$ as the number of matched observations from the pre-crisis period for stratum *s*. *w*_*j*,*s*_ is the weight assigned to pre-crisis observation *j* reflecting how often *j* is selected as control observation in the matching process and equals 1 if control observation *j* filed for insolvency at most four months after its last rating update and 0 otherwise.

Comparing actual insolvency rates with counterfactual insolvency rates for each of the sector-size strata allows us to obtain sector-size-specific estimates of the insolvency gap, *IG*_*s*_, defined as

$$ \begin{array}{@{}rcl@{}} IG_{s} = IR^{counterfactual}_{s} - IR^{actual}_{s}. \end{array} $$

**Fig. 3:**
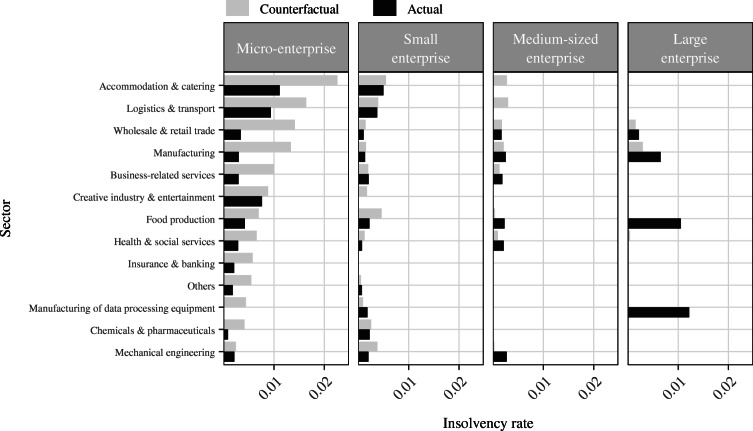
Actual and counterfactual insolvency rates. Note: The figure displays actual insolvency rates with estimated counterfactual insolvency rates for each of the *S* = 52 sector-size strata

In other words, the insolvency gap measures the extent to which observed insolvencies during the pandemic deviate from the counterfactual insolvencies that would be expected in a pre-crisis setting without policy intervention. Figure 3 contrasts actual insolvency rates against counterfactual insolvency rates and Table 6 displays the sector-size specific insolvency gap estimates along with their statistical significance. Several insights can be gained from there.

**Table 6:** Outcome analysis: insolvency gap estimation results

Sector affiliation	Size of company			
	Micro	Small	Medium	Large
	*IG*_*s*_	*IG*_*s*_	*IG*_*s*_	*IG*_*s*_
Accommodation & catering	+ 0.0115***	+ 0.0005	+ 0.0028	0.0000
Logistics & transport	+ 0.0070***	+ 0.0002	+ 0.0030	0.0000
Wholesale & retail trade	+ 0.0107***	+ 0.0004	+ 0.0001	− 0.0006
Manufacturing	+ 0.0103***	+ 0.0002	− 0.0004	− 0.0035
Business-related services	+ 0.0070***	− 0.0001	− 0.0005	0.0000
Creative industry & entertainment	+ 0.0012	+ 0.0017	0.0000	0.0000
Food production	+ 0.0027	+ 0.0024	− 0.0019	− 0.0105**
Health & social services	+ 0.0037***	+ 0.0005	− 0.0011	+ 0.0004
Insurance & banking	+ 0.0037***	0.0000	0.0000	0.0000
Others	+ 0.0037***	− 0.0002	0.0000	0.0000
Manufacturing of data processing equipment	+ 0.0044*	− 0.0009	0.0000	− 0.0122*
Chemicals & pharmaceuticals	+ 0.0033*	+ 0.0003	0.0000	0.0000
Mechanical engineering	+ 0.0003	+ 0.0018	− 0.0025***	0.0000

Note: Significance levels: *p < 0.10, **p < 0.05, ***p < 0.01. Statistical significance is based on the *χ*^2^-test for equality in the insolvency proportions in the actual and counterfactual samples using Rao-Scott corrections to the *χ*^2^ statistic (Rao & Scott, 1981) to account for the matching weights

First of all, it becomes obvious that actual insolvency rates are in almost all sectors highest among micro-enterprises (except for some outliers in the large enterprise size class). In the group of micro-enterprises, we see that actual insolvency rates are highest in the sectors which according to our survey results are also severely affected by the negative impacts of the crisis. In the accommodation and catering sector, for example, the actual insolvency rate amounts to 1.11%, in the logistics and transport sector which includes the strongly affected aviation industry we observe an insolvency rate of 0.94% and in the creative industry and entertainment sector the rate is 0.76%. These results appear intuitive and are in line with the survey results. At the same time, we find that in all sectors within the group of micro-enterprises the expected insolvency rates exceed the actual rates and in most sectors this gap is statistically significant. The average insolvency gap across all sectors in the group of micro-enterprises amounts to 0.80 pp which is substantial when being compared to the overall pre-crisis insolvency rate of 1.05%.

In the group of small enterprises, we see similar patterns although at a lower magnitude both in terms of actual insolvency rates and counterfactual rates. In fact, Table 6 suggests that the rates expected in most sectors exceed actual rates for small enterprises; however, this gap is in no sector statistically significant. On average, the insolvency gap in the group of small businesses amounts to 0.03 pp.

Moving on to the group of medium-sized enterprises, the patterns observed in the smaller size classes start to vanish. While in two of the most severely hit sectors accommodation and catering as well as logistics and transport expected insolvency rates are higher than the ones observed, the difference (i.e. the insolvency gap) is statistically not significant. For the other sectors, the picture is even more mixed. In two sectors (food production and mechanical engineering), some insolvencies took place yet almost none were predicted in the counterfactual scenario. For all other sectors, actual and counterfactual rates are very similar. Table 6 shows that none of the differences (except for the sector mechanical engineering) are statistically significant.

Ultimately, the patterns break down completely for the group of large enterprises. Barely any insolvency filing can be observed in either the crisis period or the counterfactual setting. In general, insolvencies among large corporations are rather rare events which is reflected by our results. Two sectors stand out with high actual insolvency rates: food production and manufacturing of data processing equipment. Both cases are somewhat special as they are driven by only one insolvency for which no insolvent pre-crisis control observation with comparable financial characteristics exists. Thus, one needs to be cautious when interpreting the results of the large size class.

The finding that counterfactual insolvency rates persistently, and in most sectors also significantly, exceed actual rates among micro-enterprises strongly suggests that there is a substantial backlog of insolvencies in this size class. As company size increases, the backlog of insolvencies gradually vanishes which is in line with our hypothesis that Germany’s fiscal policy response in the COVID-19 crisis disproportionately favored the survival of smaller companies. Both the temporary change in Germany’s insolvency regime and the high provision of liquidity subsidies allowed especially micro-enterprises to stay in the market. We argue that the temporary suspension of the obligation to file for insolvencies has made it particularly easy for smaller firms to use the amendment as a loophole to avert insolvency proceedings. Since disclosure requirements are more limited the smaller a company is, it becomes particularly difficult for policy makers to enforce insolvency filings among non-filing small firms. This becomes particularly problematic if the non-filing firm does not fulfill the criteria to be eligible for the suspension as it enables these companies to further absorb state subsidies. Similarly, the early on provision of direct and indirect liquidity without red tape has targeted smaller firms in particular and thus enabled them to bridge plummeting revenues in a situation in which they usually would have been forced out of the market due to illiquidity.

**Fig. 4:**
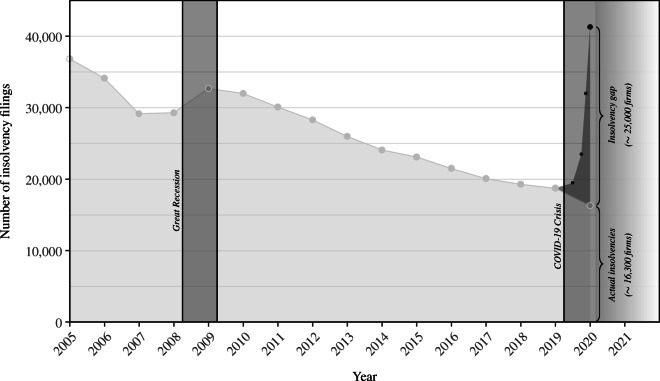
Evolution of corporate insolvencies in Germany and the COVID-19 insolvency gap. Note: The figure displays yearly absolute number of corporate insolvencies in Germany according to official statistics of the Federal Statistical Office. In 2020, both actual number of insolvencies as well as the evolution of insolvencies in the counterfactual scenario are displayed. Shaded areas reflect months of economic downturn

In order to better understand the magnitude of the insolvency gap, it is possible to aggregate and convert the insolvency gap estimates on the distinct sector-size levels into an absolute number describing the overall backlog of insolvencies (see Table 11 in the Appendix). Based on the total number of economically active companies in Germany, we estimate that the insolvency gap makes up around 25,000 companies as shown in Fig. 4. The figure reveals two further aspects. Firstly, the time series shows that during the last economic shock, the Great Recession of 2008–2009, the number of insolvencies noticeably increased, which in light of the Schumpeterian cleansing mechanism is an expected response in business dynamics. Secondly, in contrast to the Great Recession, it can be seen that in the current crisis the actual number of corporate insolvencies has declined. The observation that bankruptcy filings are lower in an economic crisis than in non-crisis times underpins that the large-scale governmental support programs have led to substantial distortions in business dynamics. Indeed, policy measures in Germany have prevented a significant number of companies from insolvency. The crucial question is, *which* firms were saved from insolvency proceedings. The following section further narrows down this question by incorporating the firms’ pre-crisis financial standing in the estimation of the insolvency gap.

#### The insolvency gap and firm viability

In order to examine whether the insolvency gap is driven by companies that are characterized by a poor financial standing before the crisis and had faced a relatively high risk of market exit when the pandemic hit, we split the sector-size strata further according to the observations’ pre-crisis financial standing. More precisely, we split each sector-size strata into two further sub-strata. The first sub-strata contains all observations whose three year average credit rating prior to the crisis is better than the overall median rating index. We refer to these as observations with ‘strong financial standing’, viable to survive the crisis based on their pre-crisis conditions. The other sub-strata comprises all observations worse than the overall pre-crisis median rating. Firms falling in such sub-strata are referred to as having a ‘weak financial standing’. Given their pre-crisis financial circumstances, we expect them to be more vulnerable to default in the current crisis or even if the pandemic had not hit the economy.

Table 7 shows the insolvency gap estimates analogous to Table 6 with the additional distinction between strata comprising financially strong companies and financially weak ones. Several aspects become apparent from these results. First, we observe that among micro-enterprises with above median credit rating (top panel) there is in almost no sector a significant deviation between actual and counterfactual insolvency rate. There are, however, two exceptions. Both in the accommodation and catering sector and the creative and entertainment sector observed insolvencies significantly exceed expected insolvencies. We know from our survey that these two sectors are by far the most affected industries. Given the severe impairments in these industries, it seems plausible that companies which had been rated relatively well before the pandemic nevertheless file for insolvency more frequently than the counterfactual estimation would suggest. This means that, despite the cushioning effect provided by fiscal policy, micro-firms with a strong pre-crisis rating filed for bankruptcy more frequently than would have been possible to learn from the financial paths of similarly strong firms in pre-crisis times. Most important, however, is the finding that among micro-enterprises the insolvency gap as backlog of expected insolvencies is *not* driven by firms with a strong financial standing prior to the crisis. In contrast, it is driven by less viable companies with a rating worse than the median rating. This results from the insolvency gap estimates among micro-enterprises with weak financial standing (bottom panel). It becomes apparent that throughout all sectors the counterfactual insolvency rates exceed the actual rates indicating a backlog of insolvencies which in the majority of sectors is not only statistically significant but in some also substantial in magnitude. In fact, the gap amounts to more than 1.70 pp in accommodation and catering, wholesale and retail as well as manufacturing which is a substantial backlog when taking into consideration that the overall pre-crisis insolvency rate lies at 1.05%.

**Table 7:** Outcome analysis: insolvency gap estimation results incorporating firms’ pre-crisis financial condition

Viability	Sector affiliation	Size of company			
		Micro	Small	Medium	Large
		*IG*_*s*_	*IG*_*s*_	*IG*_*s*_	*IG*_*s*_
Strong financial standing (pre-crisis)					
	Accommodation & catering	− 0.0029***	− 0.0013	0.0000	0.0000
	Logistics & transport	− 0.0008	− 0.0001	+ 0.0003	0.0000
	Wholesale & retail trade	+ 0.0003	− 0.0001	+ 0.0005	− 0.0007
	Manufacturing	− 0.0001	− 0.0001	− 0.0008	− 0.0038
	Business-related services	− 0.0002	− 0.0006	− 0.0010	0.0000
	Creative industry & entertainment	− 0.0025**	0.0000	0.0000	0.0000
	Food production	− 0.0007	+ 0.0020	− 0.0027*	− 0.0112*
	Health & social services	− 0.0001	− 0.0004	− 0.0003	0.0000
	Insurance & banking	+ 0.0007	0.0000	0.0000	0.0000
	Others	− 0.0002	+ 0.0002	0.0000	0.0000
	Manufacturing of data processing equipment	+ 0.0007	− 0.0015	0.0000	− 0.0127*
	Chemicals & pharmaceuticals	+ 0.0017	+ 0.0020	0.0000	0.0000
	Mechanical engineering	− 0.0003	− 0.0017	− 0.0015*	0.0000
Weak financial standing (pre-crisis)					
	Accommodation & catering	+ 0.0171***	+ 0.0018	+ 0.0030	0.0000
	Logistics & transport	+ 0.0128***	+ 0.0004	+ 0.0049	0.0000
	Wholesale & retail trade	+ 0.0196***	+ 0.0020	− 0.0035	0.0000
	Manufacturing	+ 0.0184***	+ 0.0015	+ 0.0060	− 0.0060
	Business-related services	+ 0.0122***	+ 0.0013	+ 0.0033	0.0000
	Creative industry & entertainment	+ 0.0029	+ 0.0032	0.0000	–
	Food production	+ 0.0051	+ 0.0030	+ 0.0025	0.0000
	Health & social services	+ 0.0059***	+ 0.0022	+ 0.0010	+ 0.0060
	Insurance & banking	+ 0.0055***	0.0000	0.0000	0.0000
	Others	+ 0.0073***	− 0.0012	0.0000	0.0000
	Manufacturing of data processing equipment	+ 0.0065	0.0000	0.0000	0.0000
	Chemicals & pharmaceuticals	+ 0.0050	− 0.0050	0.0000	0.0000
	Mechanical engineering	+ 0.0014	+ 0.0126*	− 0.0056	0.0000

Note: The upper panel displays insolvency gap estimates for firms with ‘strong financial standing’ comprising all firms whose three year average credit index prior to the crisis is better than the median rating index. The lower panel shows results for companies with a ‘weak financial standing’ including those with a rating worse than the median rating. For large firms in creative and entertainment sector with weak financial standing the insolvency gap could not have been calculated as no firm in this strata has been observed during the crisis period. Significance levels: *p < 0.10, **p < 0.05, ***p < 0.01. Statistical significance is based on the *χ*^2^-test for equality in the insolvency proportions in the actual and counterfactual samples using Rao-Scott corrections to the *χ*^2^ statistic (Rao & Scott, 1981) to account for the matching weights

In the small business group, we see that in the strata with strong financial performance, the size and sign of the insolvency gap estimates are comparable to the results in the micro firm group (except for accommodation/catering and creative/entertainment sector). These results indicate that there is no significant gap in insolvency filings among small businesses with above median credit rating. In the strata of small and financially weak businesses, in turn, we observe for most sectors a positive sign in the insolvency gap estimation albeit only statistically significant in the mechanical engineering sector. Again, this suggests that also in the group of small firms the backlog of insolvencies is driven by companies with weak pre-crisis conditions even if magnitude and significance is less pronounced in comparison to the micro size group.

**Fig. 5:**
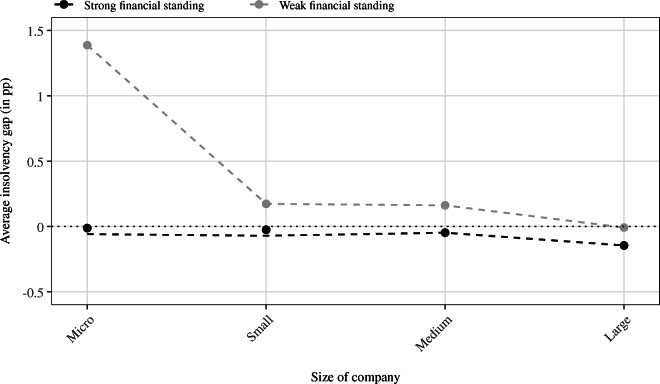
Average insolvency rates by size class and pre-crisis viability. Note: The figure displays average insolvency gaps (weighted by the number of observations falling into the matched strata) distinguishing by company size and pre-crisis financial conditions. It becomes apparent that the backlog of insolvencies is strongly driven by micro-enterprises with weak credit rating prior to the crisis and also that with increasing firm size the gap becomes less pronounced among financially weak companies

**Fig. 6:**
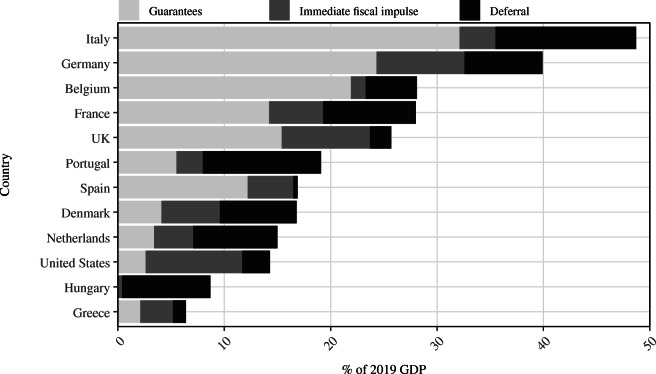
COVID-19 liquidity support through fiscal policy measures by international comparison. Note: Calculations are retrieved from Anderson et al. (2020). Numbers reflect the amount (as share of 2019 GDP) of fiscal policy measures to address adverse COVID-19 impacts on companies for selected OECD countries. Numbers are as of November 18, 2020

**Fig. 7:**
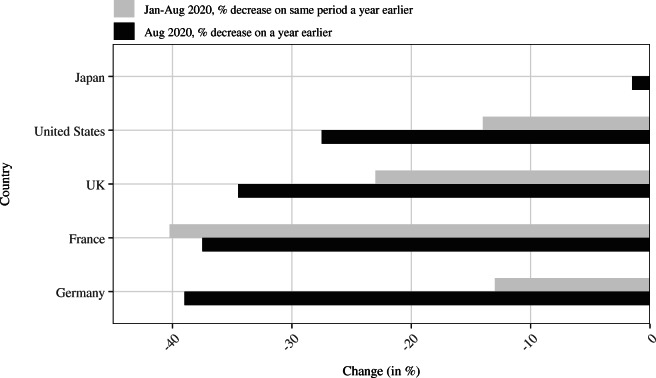
Decline in corporate insolvencies during the COVID-19 crisis. Note: The figure shows the percentage change of insolvencies in the crisis year 2020 compared to 2019 for a number of selected countries. Bar chart is adapted from The Economist (2020b)

Similar to the results in Table 6 and in line with our hypothesis that the fiscal policy response in Germany disproportionately favored survival of smaller companies, the observed patterns for small and especially micro-sized firms gradually vanish with increasing firm size as shown in Fig. 5. Consequently, the insolvency gap estimates for medium-sized and large enterprises do not reveal clear patterns in the sign of the estimates nor significant deviations between observed and predicted rates (apart for some aforementioned exceptions).

Our results show that the COVID-19-induced policy response has created a non-negligible insolvency gap that is strongly driven by micro-enterprises, which were already in a comparatively weak financial situation before the crisis.

This suggests that the early policy answer to dampen the economic impacts of the COVID-19 crisis has indeed hampered the natural cleansing effect typically observed during economic crises.

## Discussion and conclusion

The ongoing COVID-19 crisis has placed a special role on policy in order to soften the adverse economic impacts faced by many firms. There is little doubt that, in the short term, liquidity subsidies and loan guarantees have been necessary to save companies under severe liquidity pressure from insolvency. In Germany, a country where fiscal policy played a crucial role in mitigating the crisis’ impact, liquidity subsidies were accompanied by a temporary suspension of the insolvency regime. While both measures are different in design, they target the same objective: preventing an unprecedented wave of corporate insolvencies. Studying Germany’s policy response, it becomes also apparent that a number of aid schemes were either explicitly designed to save smaller companies or at least implicitly favored the survival of particularly small entrepreneurial firms. This policy environment is the basis for our hypothesis that a substantial backlog of insolvencies has accumulated particularly among SMEs as a result of the COVID-19 policy response. If, however, support schemes postpone or even prevent the exit of financially weak SMEs, there is the danger of negative long-term effects on the entire economy. In fact, in the ongoing crisis it is likely that early liquidity issues increasingly translate into an erosion of firms’ equity. Suspending bankruptcy proceedings of such over-indebted firms over a longer period of time not only is ‘to deny reality’ (The Economist 2020a, 3) but also hampers the efficient reallocation of resources. In this vein, economic crises also serve as cleansing mechanism to release resources from inefficient and non-innovative firms which typically find more productive use elsewhere. The early policy response of the German government not only has been targeted disproportionately at smaller firms but also did so with little screening mechanisms in place (see, for example, Federal Ministry for Economic Affairs and Energy 2020) rescuing companies from insolvency in a fairly indiscriminate manner.

Making use of both survey data and a unique and large dataset of firm-specific credit rating data along with information on firm insolvency filings, we investigate whether the German policy response has indeed caused distortions in the natural cleansing mechanism typically encountered in liquidity crises. While the policy response to the economic impact of COVID-19 in Germany suggests notable differences in firm size, our survey results reveal strong heterogeneity across economic sectors in their exposure to the adverse effects in the current crisis. With these findings, we estimate the extent of an insolvency gap, defined as the deviation of observed insolvency rates during the COVID-19 pandemic and expected insolvency rates based on a counterfactual pre-crisis setting with no policy intervention, for 52 distinct sector-size strata. In line with our hypothesis, our results show that the insolvency gap is particularly significant in the group of micro-enterprises (at most 10 employees) and that the gap gradually vanishes with increasing firm size. Furthermore, we distinguish between financially strong and financially weak firms in our analysis with the latter being defined as companies with below median credit rating prior to the crisis. Thus, we refer to financially weak firms as companies being relatively more vulnerable to default in the current crisis based on their pre-crisis financial standing. Our findings suggest that the backlog of insolvencies is mainly driven by firms with a relatively poor credit rating prior to the crisis. This indicates that particularly financially weak, small firms may take advantage of the less stringent screening processes associated with many of the COVID-19-related policy instruments or absorb the liquidity injections as windfall gains, especially during the first months of the crisis when eligibility criteria were low.

From a welfare perspective, this comes at the burden of high fiscal costs that are associated with granting financial aid to unviable firms. Favoring the survival of financially weak firms as our findings indicate, however, also imposes indirect costs in the longer term as such firms tie up resources whose efficient redistribution would have facilitated entrepreneurship. Past experience shows that keeping distressed firms alive may severely obstruct business dynamism and structural change. Literature on Japan (Caballero et al., 2008), but also on other OECD economies (Adalet McGowan et al., 2018), suggests that granting life-sustaining credit to near-insolvent firms has not only lowered aggregate productivity but also deterred market entry of new entrepreneurs. Although in these cases the survival of insolvent firms is mostly attributed to questionable bank lending practices and not to a crisis-related policy response, some lessons can still be learned from these experiences: keeping unviable firms alive causes severe market congestion which creates barriers to market entry and limits the growth of young companies. The persistence of crisis-induced SME support along with a further prolongation of the (at least partial) moratorium of Germany’s insolvency regime increasingly favors such a market congestion with the risk of creating barriers to entrepreneurship. It is likely that once the policy instruments will cease, i.e. liquidity support will terminate and the German insolvency regime returns back to the filing obligation, a number of small business insolvencies will follow. Without an ‘evergreening’ of policy support it is, however, doubtful if they can be prevented at all. In the ongoing crisis, it will therefore become increasingly important to think about policy measures that remove entry barriers for young and innovative businesses and create growth opportunities for firms which respond innovatively to the pandemic instead of prolonging the survival of near-insolvent firms. For example, policy makers are well advised to consider law reforms that lower the barriers to corporate restructuring for viable smaller firms while streamlining and encouraging liquidation procedures for unviable companies. Past experience suggests that this would stimulate the reallocation of capital to more productive entrepreneurial endeavors (Adalet McGowan et al., 2018).

Understanding the effects of the interplay between liquidity support on the one hand and temporary adjustments to insolvency regimes on the other hand will be an important lesson from the COVID-19 crisis. Does the interplay of these two instruments impair entrepreneurship and economic recovery as it primarily discourages struggling firms from exiting the market or does it, if well dosed, even serve as a useful policy mix in liquidity crises? Our results which only look at the early policy effects in the pandemic suggest the former. It is left to future research to investigate the long-term5 effects on productivity, innovation and entrepreneurship induced by the policy responses to COVID-19.

## Figures and Tables

**Fig. 8:**
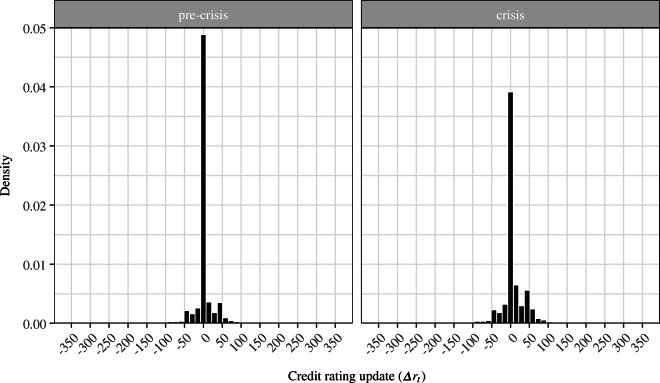
Distribution of credit rating update in pre-crisis and crisis period. We define a rating update as a reassessment of the company’s creditworthiness performed by Creditreform. We have precise information on the date of reassessment, which allows us to accurately assign the update to either the pre-crisis or the crisis period and also to accurately match the updates with insolvency dates. It should also be noted that a reassessment does not necessarily lead to a change in the rating index. If the creditworthiness of the company has not changed since the last rating, the company gets assigned the same index as before, resulting in a value of 0 in Δ*r*_*t*_. Form the figure it becomes apparent that there is a rightward shift in the distribution of rating updates during the crisis period, indicating that there were more credit rating downgrades as compared to the pre-pandemic period. This reflects that the financial situation deteriorated for a larger share of companies in the crisis period than in the three years preceding the crisis

## Data Availability

The data that support the findings of this study are deposited in the ZEW Research Data Center (ZEW-FDZ) and will be made available upon reasonable request from the corresponding author (J.O. Dörr).

## Appendix

**Table 8:** Mapping firm characteristics to size group

	Size of company			
	Micro	Small	Medium	Large
Number of employees	≤ 10	11–49	50–249	≥ 250
Annual turnover (in M €)	≤ 2	2–10	10–50	> 50
Annual balance sheet total (in M €)	≤ 2	2–10	10–43	> 43

Note: The table shows translation of firm characteristics into company size classes used in this study as defined by European Commission (2003)

**Table 9:** Mapping EU NACE Revision 2 divisions to sector groups

Sectors	Divisions
Business-related services	58–63, 68, 69–82
Manufacturing	5–9, 12–19, 23–25, 27,
	31–33, 35–39, 41–43
Wholesale & retail trade	45–47
Health & social services	86–88, 94–96
Insurance & banking	64–66
Accommodation & catering	55, 56
Logistics & transport	49–53
Creative industry & entertainment	90–93
Mechanical engineering	28–30
Food production	10, 11
Chemicals & pharmaceuticals	20–22
Manufacturing of data processing equipment	26
Others	Any division not listed above

Note: The table shows translation of EU’s NACE Revision 2 divisions (European Union, 2006) into sector groupings used in this study

**Table 10:** COVID-19 first-round policy measures in Germany: Overview

Instrument	Description	Scope	Target group	Suggested effect on
			(by firm size)	insolvency filings
Liquidity support				
Direct liquidity	‘Soforthilfen’: fully	Up to €15k per month		Weakly lowering short-term
subsidy	subsidized payments	€50bn overall		insolvency risk, weakly
	over 3 months			favoring insolvency gap
	‘Überbrückungshilfen’:	Up to 80% of fixed costs		Lowering short-term insolvency
	fully subsidized payments	(max. €50k) per month		risk, moderately favoring
	over 3 months	€25bn overall		insolvency gap
Liquidity	‘KfW-Schnellkredite’:	Up to 3 monthly turnovers		Lowering mid-term insolvency
loan under	low-interest loans hedged	(max. €800k) in total		risk, favoring insolvency gap
public guarantee	by a 100% guarantee from			
scheme	the Federal Government			
Labor cost	‘Kurzarbeitergeld’: public	Up to 87% of last net		Lowering mid-term insolvency
subsidies	wage compensations for	income for up to 21 months		risk, favoring insolvency gap
	employees’ reduced	(including social security		
	working hours if at least	charges) per employee		
	10% of workforce in			
	short-time			
Intertemporal	Various tax-related	€250bn overall		Weakly lowering mid-term
liquidity support	deferrals	(estimated)		insolvency risk, weakly
				favoring insolvency gap
Change in insolvency regime				
Temporary	‘German COVID-19	Full suspension until		No effect on actual insolvency
suspension	Insolvency Law Amendment’:	September 30, 2020		risk eventually giving the firm
of the obligation	Temporarily releases from	Suspension until January		time to take up liquidity support
to file for	the legal obligation to	31, 2021 in case of		and to make arrangements for
insolvency	disclose insolvency in case	over-indebtedness		their financing and restructuring
	of (1) iliquidity, (2)			with its creditors, strongly
	imminent iliquidity or			favoring insolvency gap
	(3) over-indebtedness			

Note: The table provides an overview of the early policy measures to support companies depending on the size of the company. Only the most important first-round policy instruments which are likely to have an impact on corporate insolvencies are presented

Size classes:  micro-enterprise,  small enterprise,  medium-sized enterprise,  large enterprise

**Table 11:** Calculation of the insolvency gap in absolute terms

Sector	Size of company						$\sum $
	Micro		Small		Medium		
	*N*_*s*_	*I**G*_*s*_ (in %)	*N*_*s*_	*I**G*_*s*_ (in %)	*N*_*s*_	*I**G*_*s*_ (in %)	
Accommodation & catering	37,633	0.0115	4,852	0.0005	810	0.0028	
Creative industry & entertainment	16,057	0.0012	1,910	0.0017	476	0.0000	
Food production	8,191	0.0027	3,674	0.0024	1,962	− 0.0019	
Health & social services	69,029	0.0037	12,331	0.0005	4,269	− 0.0011	
Insurance & banking	46,670	0.0037	2,583	0.0000	1,290	0.0000	
Logistics & transport	43,899	0.0070	10,756	0.0002	2,773	0.0030	
Chemicals & pharmaceuticals	5,170	0.0033	3,980	0.0003	2,342	0.0000	
Manufacturing of data proc. eq.	4,270	0.0044	2,449	− 0.0009	1,057	0.0000	
Mechanical engineering	10,567	0.0003	6,828	0.0018	3,386	− 0.0025	
Business-related services	287,115	0.0070	40,448	− 0.0001	9,871	− 0.0005	
Manufacturing	251,027	0.0103	50,447	0.0002	12,399	− 0.0004	
Others	37,695	0.0037	5,381	− 0.0002	2,398	0.0000	
Wholesale & retail trade	201,838	0.0107	46,342	0.0004	10,549	0.0001	
Weighted insolvency gap (in %)	0.0080		0.0003		− 0.0003		
Number of active firms (official statistics)	3,109,261		293,610		63,928		3,466,799
Insolvency gap (absolute)	24,933		90		− 19		25,004

Note: Weighted insolvency gap of each size class is calculated as average of the sector specific insolvency gap estimates weighted by the number of observations of the overall sample in the respective stratum. Number of active firms in Germany reflect the latest official statistics of the https://www-genesis.destatis.de/genesis//online?operation=table&code=52111-0001&bypass=true&levelindex=0&levelid=1615296169401. Insolvency gap in absolute terms is calculated as product between the weighted insolvency gap and the total number of active German firms within the respective size class. Due to the small number of large firm insolvencies, we refrain from converting the estimates into absolute numbers in this size class

**Table 12:** Improvement in balance through matching

Sector	Size	% Improvement in eCDF mean					Variance ratio				
		Δ*r*_*t*_	*r*_*t*−*x*_	$\bar {r}_{t}$	*d*_*t*_	*a*_*t*_	Δ*r*_*t*_	*r*_*t*−*x*_	$\bar {r}_{t}$	*d*_*t*_	*a*_*t*_
Accommodation & catering	Micro	97	89	90	96	71	1.00	1.01	1.01	1.01	1.38
	Small	96	47	45	67	− 15	1.00	1.10	1.11	1.04	1.66
	Medium	96	6	6	− 16	− 119	1.00	1.25	1.26	1.10	2.26
	Large	91	22	3	47	− 71	1.13	0.71	0.67	1.31	6.59
Business-related services	Micro	95	91	92	99	89	1.01	1.01	1.01	1.00	1.02
	Small	88	57	72	95	81	1.02	1.05	1.06	1.01	1.09
	Medium	83	78	84	88	65	1.02	1.06	1.09	1.01	1.14
	Large	74	22	− 2	47	25	1.04	1.09	1.12	1.05	1.21
Chemicals & pharmaceuticals	Micro	81	40	41	19	40	1.01	1.09	1.12	1.04	1.09
	Small	74	47	64	83	47	1.02	1.07	1.10	1.06	1.16
	Medium	81	14	63	66	− 13	1.03	1.14	1.13	1.09	1.22
	Large	74	− 30	1	3	11	1.01	1.23	1.23	1.18	1.22
Creative industry & entertainment	Micro	95	71	76	59	36	1.01	1.03	1.03	1.02	1.09
	Small	95	53	26	65	23	1.01	0.99	0.97	1.08	1.26
	Medium	94	− 71	− 64	− 3	− 79	1.03	1.29	1.33	1.11	2.15
	Large	87	1	19	87	− 19	1.07	0.81	0.72	1.05	0.60
Food production	Micro	85	77	79	67	32	1.01	1.04	1.03	1.04	1.16
	Small	83	36	42	89	− 28	1.00	1.03	1.03	1.02	1.18
	Medium	71	− 50	− 19	12	− 58	1.03	1.28	1.29	1.07	1.14
	Large	62	55	47	33	34	1.00	1.48	1.48	1.22	1.02
Health & social services	Micro	95	89	91	92	80	1.01	1.01	1.01	1.01	1.18
	Small	89	47	50	25	35	1.02	1.05	1.05	1.03	1.17
	Medium	84	42	24	78	30	1.01	1.12	1.12	1.08	1.11
	Large	71	54	39	79	− 39	1.03	1.24	1.27	1.10	1.44
Insurance & banking	Micro	90	86	88	89	80	1.01	1.02	1.02	1.00	1.05
	Small	73	49	67	82	73	1.03	1.04	1.04	1.07	1.07
	Medium	52	61	50	92	63	1.04	1.05	1.07	1.00	1.06
	Large	79	59	59	96	71	1.08	1.17	1.16	1.02	0.98
Logistics & transport	Micro	93	87	87	93	62	1.01	1.02	1.02	1.01	1.05
	Small	89	− 27	49	0	53	1.02	1.12	1.13	1.04	1.09
	Medium	84	− 9	35	52	11	1.02	1.14	1.16	1.07	1.19
	Large	85	− 78	− 107	77	− 9	1.05	1.30	1.26	1.09	1.26
Manufacturing	Micro	93	92	93	97	82	1.01	1.01	1.01	1.00	1.03
	Small	87	72	80	99	68	1.02	1.04	1.05	1.00	1.04
	Medium	84	− 2	54	67	37	1.02	1.09	1.11	1.03	1.07
	Large	85	− 27	19	73	− 23	1.03	1.23	1.20	1.12	1.25
Manufacturing of data processing equipment	Micro	74	− 20	0	89	44	1.01	1.06	1.05	1.05	1.19
	Small	71	19	43	81	32	1.01	1.20	1.16	1.07	1.28
	Medium	73	− 31	23	− 35	45	1.05	1.55	1.58	1.11	1.27
	Large	79	32	56	65	3	1.04	1.11	1.04	1.17	1.60
Mechanical engineering	Micro	83	27	31	92	45	1.01	1.07	1.06	1.01	1.11
	Small	77	− 9	37	89	25	1.02	1.10	1.13	1.02	1.12
	Medium	85	− 22	37	78	− 7	1.01	1.22	1.18	1.05	1.19
	Large	87	3	18	54	28	1.02	1.30	1.39	1.08	1.16
Others	Micro	93	88	90	91	64	1.01	1.01	1.02	1.01	1.12
	Small	86	32	52	93	35	1.02	1.02	1.04	1.01	1.26
	Medium	84	45	63	72	42	1.03	1.08	1.08	1.02	1.05
	Large	74	25	48	80	− 47	1.01	1.08	1.06	1.12	1.11
Wholesale & retail trade	Micro	96	91	92	98	82	1.01	1.01	1.01	1.00	1.03
	Small	92	11	49	93	67	1.01	1.04	1.04	1.01	1.04
	Medium	87	33	66	82	42	1.02	1.11	1.11	1.02	1.10
	Large	83	30	45	75	20	1.03	1.18	1.17	1.08	1.15

Note: The table shows balance assessment statistics for all matching variables. % improvement in empirical cumulative density function (eCDF) mean shows by how much percent the deviation in the eCDF mean between pre-crisis and crisis observations has improved through nearest neighbor matching. It becomes apparent that for most covariates in all sector-size strata a substantial improvement in balance has been achieved through the matching process. Variance ratio statistics refer to the ratio of the variance among the matched control observations and the variance among the crisis observations for the respective variable. Values closer to zero indicate better balance in variance

## Footnotes

1 In Germany, for instance, liquidity grants’ ‘application and payment process is to be swift and free from red tape’ according to the Ministry of Finance (Federal Ministry of Finance 2020b, para. 2). Moreover, in context of public loan programs it is stated that ‘the credit approval process does not involve additional credit risk assessment by the bank’ and that ‘there are no requirements for collateral security’ (Federal Ministry for Economic Affairs and Energy 2020, para. 5).

2 The surveys have been conducted in April 2020, in June 2020 and in September 2020 spanning the period of the full suspension of the obligation to file for insolvency and is therefore particularly suitable for capturing the early policy-induced effects of the crisis.

3 0 indicates no negative effects, 4 signals strong negative effects.

4 In our analysis a company is defined as economically active if it has received a credit rating update at least once over the last three years spanning the period from July 2017 to July 2020.

5 We observe one and the same company at most three times in our sample. Thus, credit rating updates normally do not take place more often than once per year but may be conducted in a less regular cycle.

6 Note that we exclude observations between January 2020 and March 2020 which we see as transitional phase in which assignment to either of the two periods is not straightforward. Also note that July 2020 is the latest month for which we observe credit rating information.

7 Figure 2 provides an illustration of how closely matching observations from the pre-crisis period serve as controls for rating changes of firms in the crisis period.

8 The credit rating index suffers from a discontinuity as in case of a ‘insufficient’ creditworthiness it takes on a value of 600 (Creditreform, 2020a). We truncate credit ratings of 600 to a value 500—the worst possible rating in our analysis. We do so since our main predictor variable is the *update* in the rating index which can only be reasonably calculated if the index has continuous support.

9 Reassessments of the rating is conducted in an irregular fashion such that the time between two updates, *x*, varies. On average, the time between two updates equals 20 months.

10 Note that we define a rating update as a reassessment of the company’s creditworthiness performed by Creditreform. We have precise information on the date of reassessment, which allows us to accurately assign the update to either the pre-crisis or the crisis period and also to accurately match the updates with insolvency dates. It should also be noted that a reassessment does not necessarily lead to a change in the rating index. If the creditworthiness of the company has not changed since the last rating, the company gets assigned the same index as before, resulting in a value of 0 for Δ*r*_*t*_.

11 For example, for a credit rating observation in July 2017, we count how often the firm experienced a downgrade over the period June 2014 to June 2017 and also calculate the average rating over that period.

12 See also Fig. 8 in the Appendix for a comparison of the distribution of the credit rating updates in the pre-crisis and the crisis period.

13 *s* ∈ [1, 52].
